# Supplementary material for: In-Depth Annotation of the Drosophila Bithorax-Complex Reveals the Presence of Several Alternative ORFs That Could Encode for Motif-Rich Peptides
Source: Cells. 2021 Nov 2;10(11):2983. doi: 10.3390/cells10112983 (PMC8616405; doi:10.3390/cells10112983)
Supplement: Supplementary file 1 [file cells-10-02983-s001.zip › cells-1399916-SI/cells-1399916-sup fig.pdf]

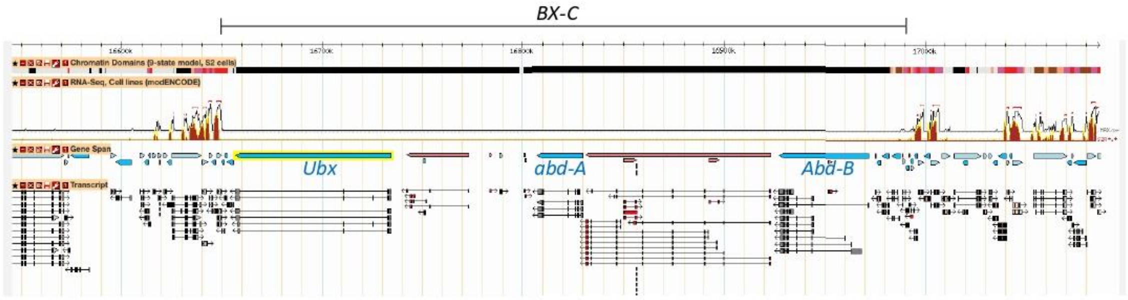

Supplementary Figure S1. Genome region covering BX-C and depicting the binding of the repressive state chromatin protein Polycomb (Pc, black bar) and RNA-seq (red peaks) in S2 cells. Scheme adapted from Flybase (http://flybase.org/).

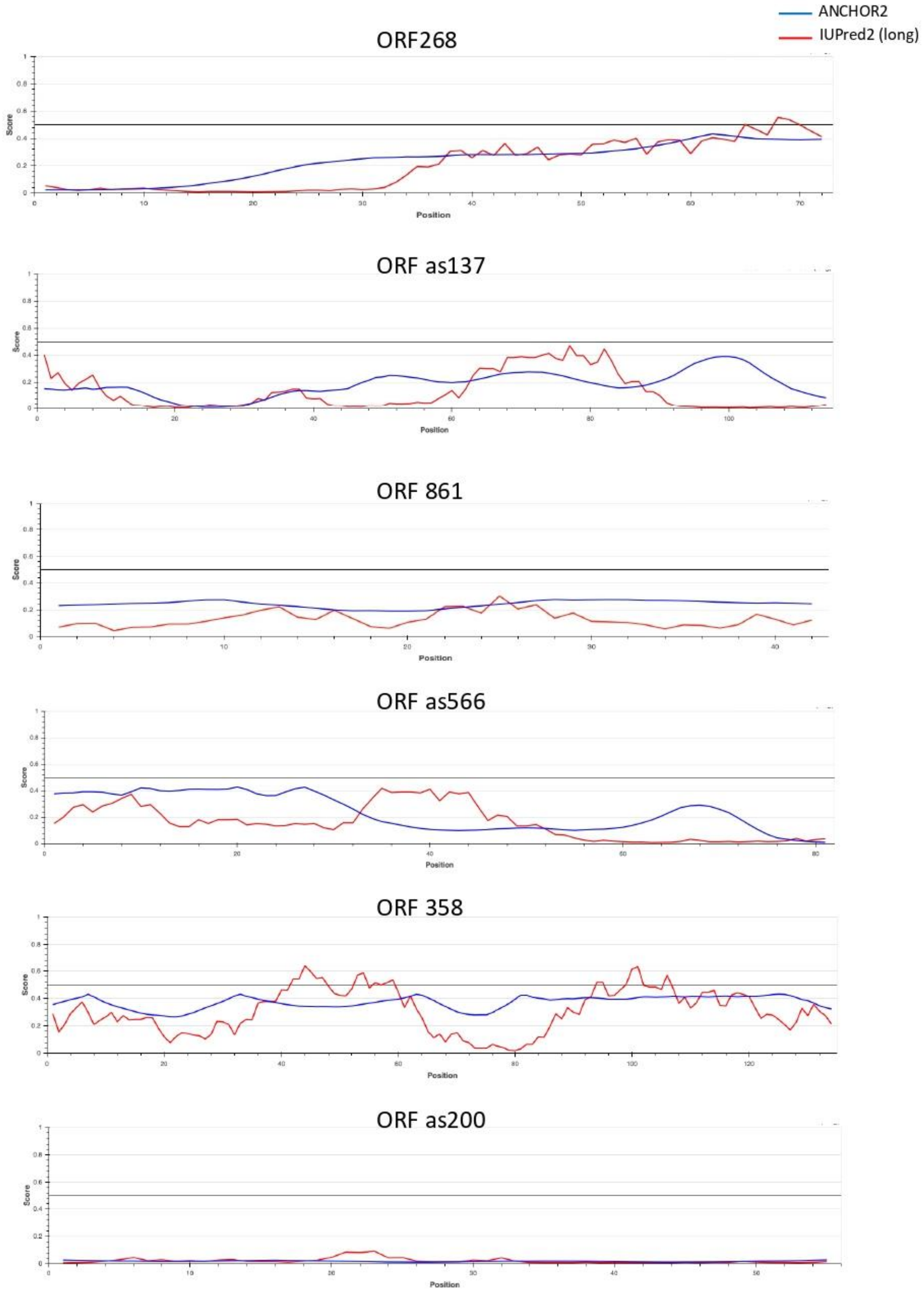

Supplementary Figure S2. Examples of IUPred profiles of globally ordered altORFs.

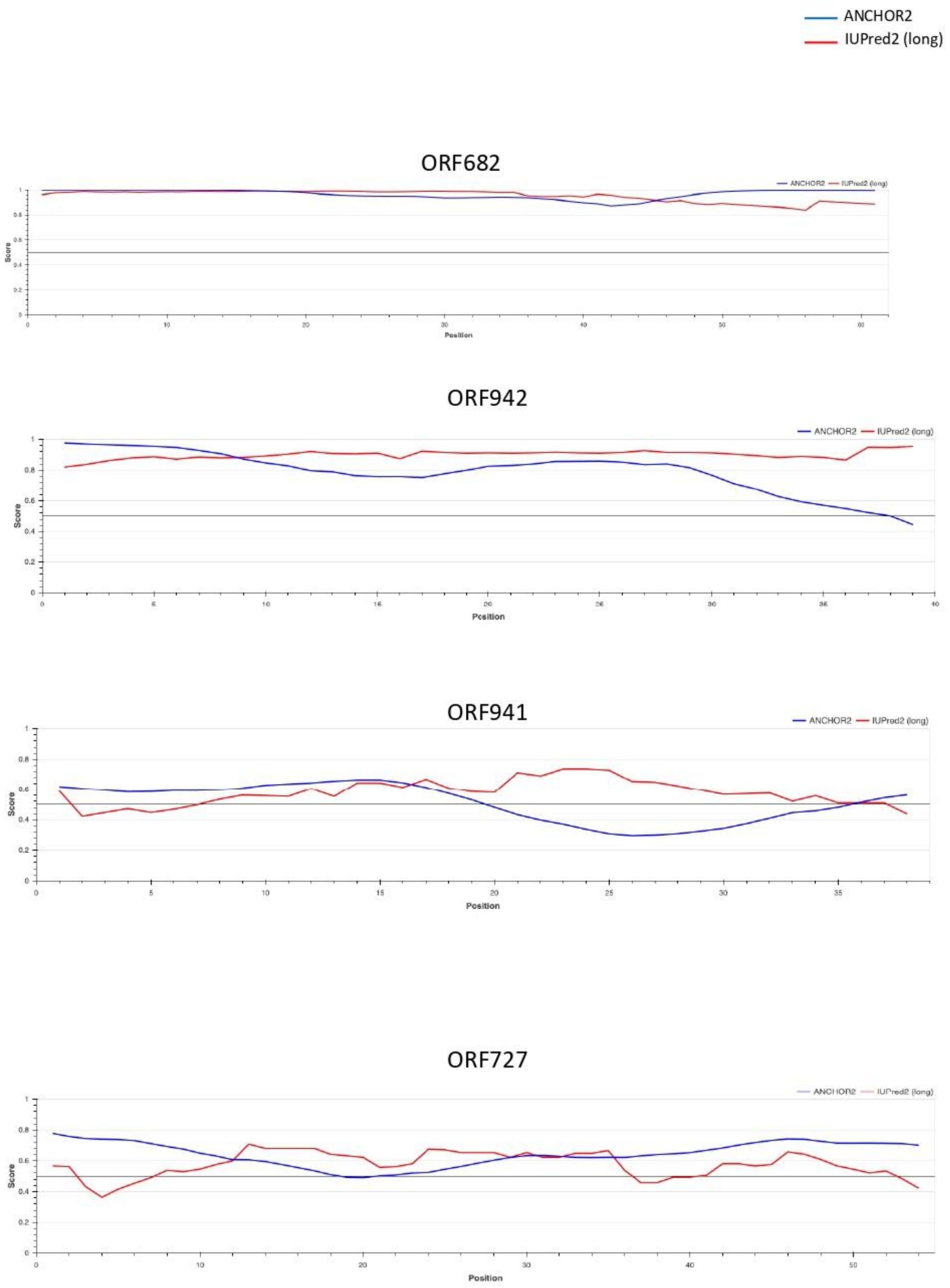

Supplementary Figure S3. Examples of IUPred profiles of globally disordered altORFs.

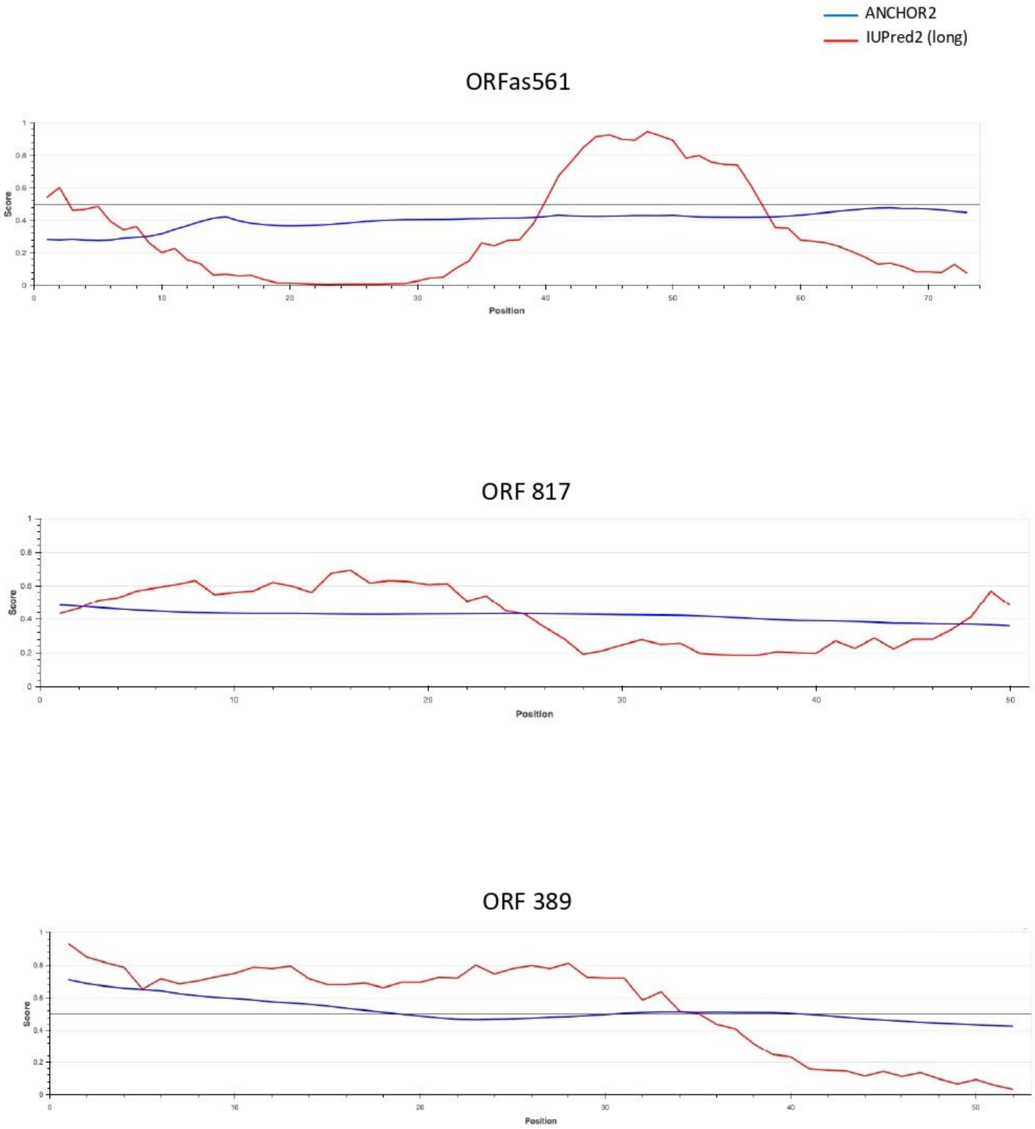

Supplementary Figure S4. Examples of IUPred profiles of altORFs displaying both ordered and disordered regions.

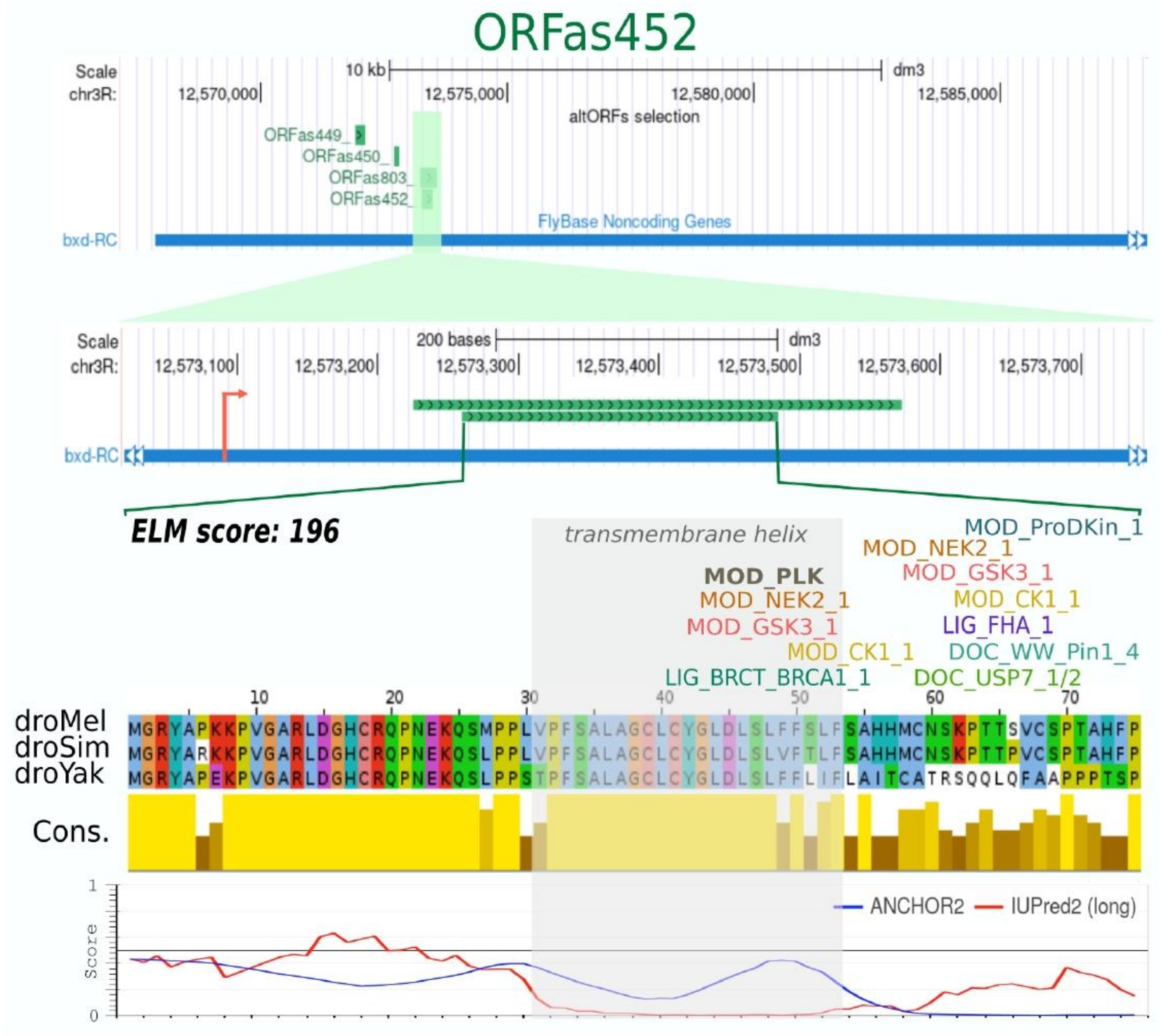

Supplementary Figure S5. Characterization of the ORFas452. This altORF is located in the *bxd-RC* lncRNA, in opposite orientation. It is found in 3 *Drosophila* species and is highly conserved (yellow bars below the protein sequences). The 75 aas long predicted protein is globally ordered and has a transmembrane helix and several ELMs of different types.

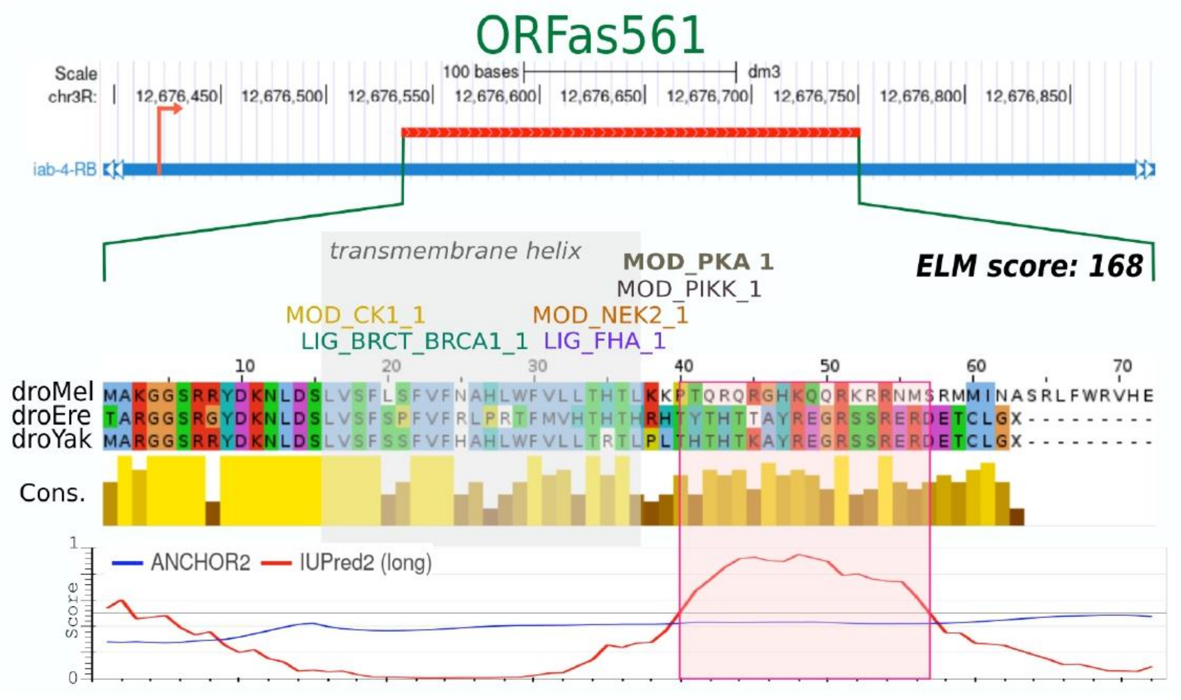

Supplementary Figure 6

Supplementary Figure S6. Characterization of the ORFas561. This altORF is located in the *iab-4-RB* lncRNA, in the same orientation. It is found in 3 *Drosophila* species. The 72 aas long predicted protein has both ordered and disordered domains, a transmembrane helix domain and several ELMs of different types.

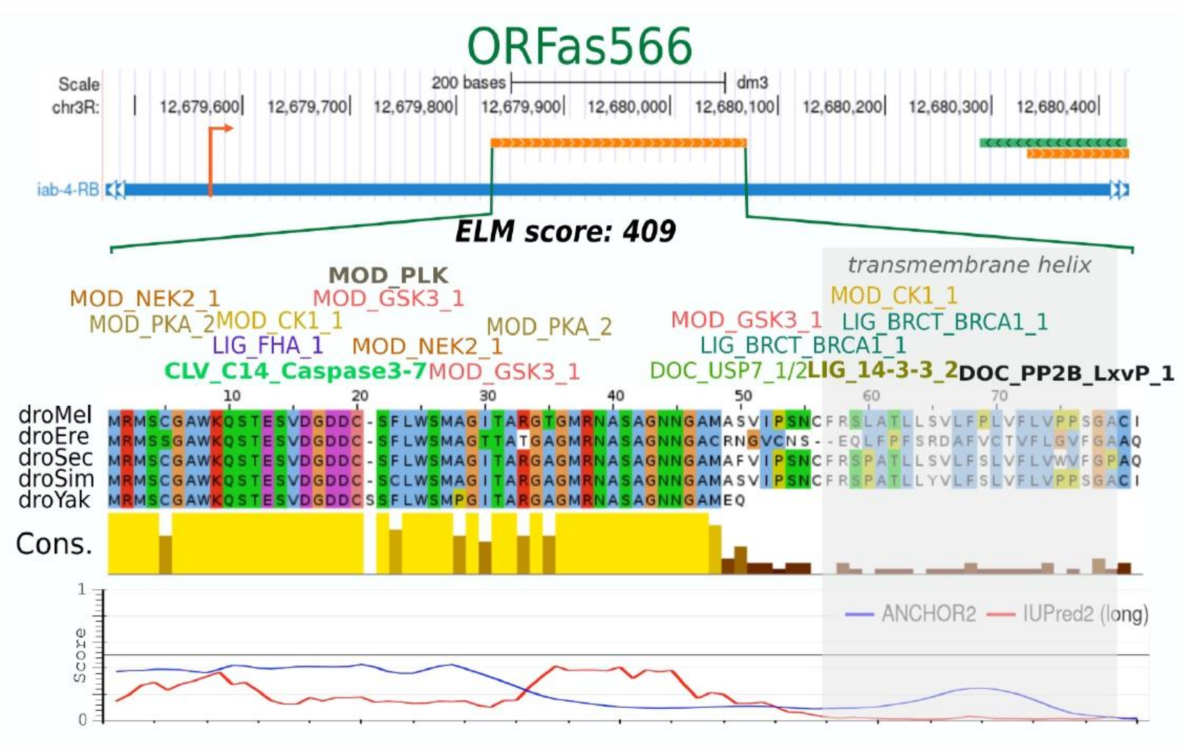

Supplementary Figure S7. Characterization of the ORFas566. This altORF is located in the *iab-4-RB* lncRNA, in the same orientation. It is found is 2 *Drosophila* species. Its 5′ half is highly conserved, in contrast to its 3′ half. The 80 aas long predicted protein is globally ordered and presents a transmembrane helix (in the less conserved region) and a number of ELMs of different types.

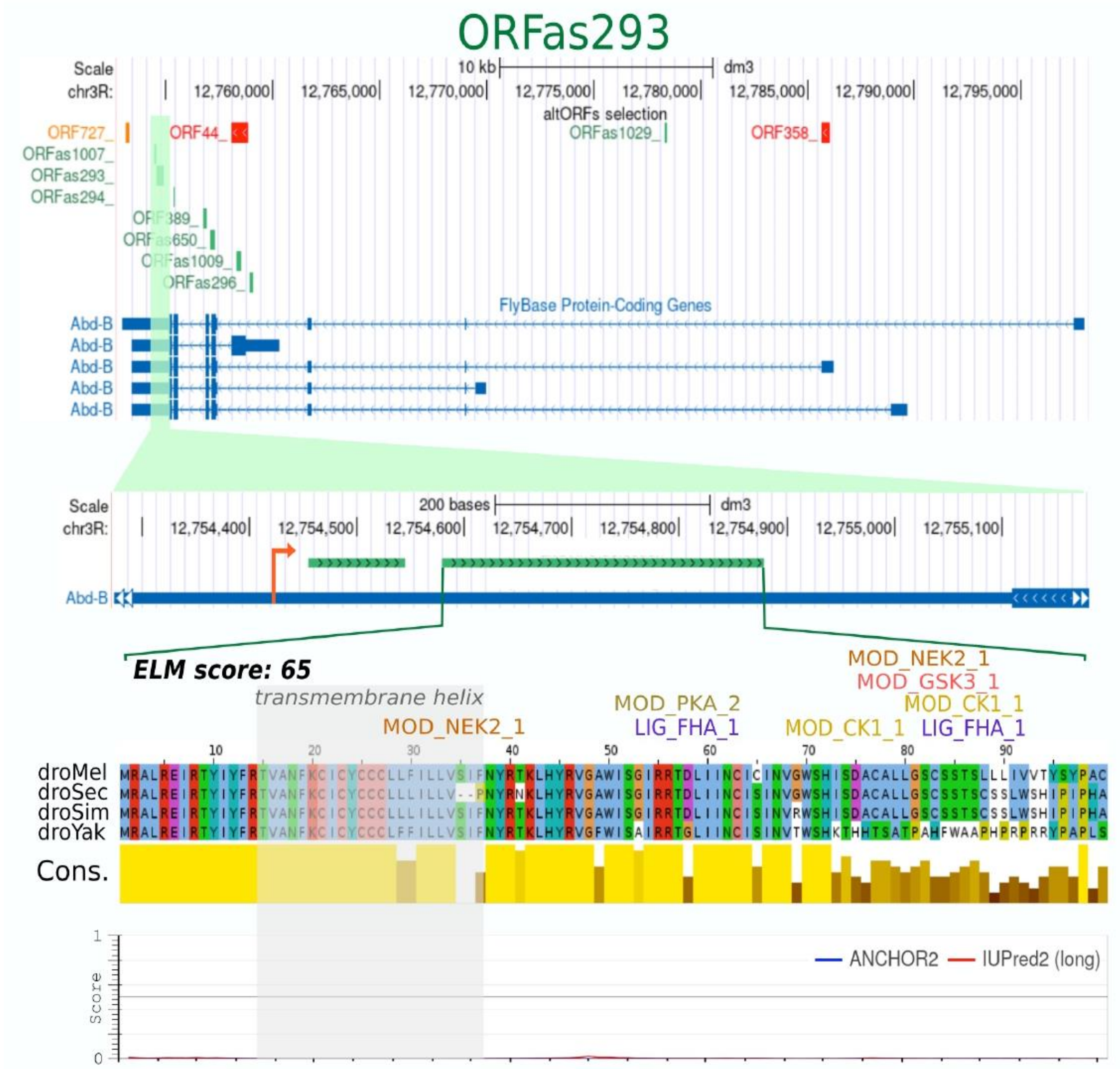

Supplementary Figure S8. Characterization of the ORFas293. This altORF is located in the *Abd-B* 3'UTR, in opposite orientation. It is found in 4 *Drosophila* species and is highly conserved over the first two thirds of its length. The 100 aas long predicted protein is highly ordered and has a transmembrane helix (in the conserved part) and several ELMs of different types.
